# Myeloid-Derived Suppressor Cell Differentiation in Cancer: Transcriptional Regulators and Enhanceosome-Mediated Mechanisms

**DOI:** 10.3389/fimmu.2020.619253

**Published:** 2021-01-14

**Authors:** Norman Fultang, Xinyuan Li, Ting Li, Youhai H. Chen

**Affiliations:** Department of Pathology and Laboratory Medicine, Perelman School of Medicine, University of Pennsylvania, Philadelphia, PA, United States

**Keywords:** myeloid-derived suppressor cell, immunosuppression, enhanceosome, aberrant myelopoiesis, tumor immunobiology

## Abstract

Myeloid-derived Suppressor Cells (MDSCs) are a sub-population of leukocytes that are important for carcinogenesis and cancer immunotherapy. During carcinogenesis or severe infections, inflammatory mediators induce MDSCs *via* aberrant differentiation of myeloid precursors. Although several transcription factors, including C/EBPβ, STAT3, c-Rel, STAT5, and IRF8, have been reported to regulate MDSC differentiation, none of them are specifically expressed in MDSCs. How these lineage-non-specific transcription factors specify MDSC differentiation in a lineage-specific manner is unclear. The recent discovery of the c-Rel−C/EBPβ enhanceosome in MDSCs may help explain these context-dependent roles. In this review, we examine several transcriptional regulators of MDSC differentiation, and discuss the concept of non-modular regulation of MDSC signature gene expression by transcription factors such as c-Rel and C/EBPß.

## Introduction

Tumor immune evasion is an essential feature of tumorigenesis (1, 2). To successfully establish themselves within a host, tumor cells leverage biochemical signals and rogue immune cells to hide from and repress host immune responses (1–3). Immunotherapy, which can restore immune response and anti-cancer immunity, has revolutionized cancer therapy. However, rogue immunosuppressive cells, including tumor-associated macrophages (TAMs), tumor-associated neutrophils (TANs), regulatory T-cells (Tregs), regulatory dendritic cells (RegDCs), cancer-associated fibroblasts, and myeloid-derived suppressor cells (MDSCs), still represent significant impediments to immunotherapy, contributing to therapy failure and poor clinical outcomes (4–8). Of these pro-tumoral cell types, MDSCs are perhaps the least well characterized.

MDSCs are a heterogenous population of immunosuppressive pro-tumoral leukocytes which arise as a result of defects in myelopoiesis (9). Under physiological conditions, progenitor myeloid cells differentiate into macrophages, dendritic cells or granulocytes. Under pathological conditions like cancer or chronic infections, aberrant myelopoiesis allows the accumulation and expansion of immature myeloid cells with strong immunosuppressive capabilities (10–16). While these cells possess many phenotypic and morphological hallmarks of anti-tumor myeloid-lineage cells like monocytes and neutrophils, they differ significantly in their activation programs and function to inhibit anti-tumor immunity by producing immunosuppressive factors like arginase, nitrogen species and reactive oxygen species, among others (10, 17–19). MDSCs are a significant obstacle to immunotherapies including checkpoint inhibitors; accumulation of MDSCs populations within circulating and tumor-infiltrating leukocytes have been observed in patients who fail to respond to checkpoint inhibitor therapy (18, 19).

There are two major subsets of MDSCs– granulocytic or polymorphonuclear MDSCs (G-MDSCs or PMN-MDSCs), which are phenotypically similar to granulocytes, and monocytic or mononuclear MDSCs (M-MDSCs), which are phenotypically similar to monocytes. PMN-MDSCs have a CD11b^+^Ly6G^+^Ly6C^lo^ phenotype in mice and a CD11b^+^CD14^−^CD15^+^/CD66b^+^ phenotype in humans while M-MDSCs are identified as CD11b^+^Ly6G^−^Ly6C^hi^ in mice, and CD11b^+^CD14^+^HLA^-^DR^−/lo^CD15^−^ in humans (20, 21). MDSC markers were recently reviewed here (21). A third mixed population of MDSCs, early-stage MDSC (e-MDSC), with phenotype Lin^-^ (including CD3, CD14, CD15, CD19, and CD56) HLA^-^DR^-^CD33^+^ was recently proposed in humans (22). e-MDSCs also contain immature progenitor myeloid cells and their equivalent in mice is yet to be identified (22).

While a lot is known about the phenotypic and morphological delineations of MDSCs, the biochemical markers and effectors underlying their development and function are still poorly understood. As such, the identification of these drivers of pathological MDSC expansion and immunosuppressive activity has been the subject of intensive research in recent years. Recently identified MDSC effectors, mostly transcription factors (TFs) and apoptotic regulators, include IRF8 (23), STAT3 (23–26), C/EBPß (27, 28), S100A8/9 (29), TIPE2 (30, 31), GCN2 (32), among others (**Table 1**). Of all these regulators, C/EBPß has emerged as an essential “master” regulator of MDSC expansion and immunosuppressive activity. Most of the known MDSC regulators drive expansion and immunosuppressive activity in C/EBPß-dependent mechanisms. Additionally, C/EBPß deletion alone in myeloid cells was sufficient to halt MDSC generation and immunosuppressive activity (27). Recent evidence, however, suggests that c-Rel, a member of the NF-κB (nuclear factor kappa-light-chain-enhancer of activated B cells) family of transcription factors, regulates C/EBPß activity and expression in MDSCs (33). In this review we describe c-Rel and C/EBPß as master effectors of MDSC biology and highlight how a non-modular c-Rel-C/EBPß “enhanceosome” drives MDSC development and function in cancer.

**Table 1:** Known effectors or regulators of MDSC biology.

Effectors	Mechanisms	References
STAT3	Stimulates inflammatory cytokines, activates transcription of immunosuppressive enzymes with C/EBPß. Downregulates IRF8	(23, 33, 34)
STAT5	Downregulates IRF8, promoting aberrant myeloid differentiation	(23)
C/EBPß	Master regulator. Promotes transcription of immunosuppressive enzymes and inflammatory cytokines in tumor microenvironment	(27, 35–37)
IRF8	Crucial for normal myeloid differentiation. Negative regulator of MDSCs. Downregulated by STAT3/5	(23)
S100A8/9	Produced by tumors. Binds to RAGE receptors in myeloid precursors and activates immunosuppressive NF-κB-C/EBPß-STAT3 signaling axis.	(29)
RB	Epigenetically silenced by HDAC6 in MDSCs. Negatively regulates myeloid differentiation into PMN-MDSCs.	(38)
TIPE2	Induced by IL-6 and high ROS in tumor microenvironment. Activates C/EBPß and STAT3 which promote immunosuppressive activity.	(30, 31)
GCN2	Polarity switch. Expression correlates with immunosuppressive activity. Induces C/EBPß and CREB2/ATF4 promoting immunosuppression.	(31)

## Known MDSC Effectors

MDSCs arise when sustained pathologic inflammation induces an aberrant differentiation program in myeloid precursors giving rise to immunosuppressive cells (10–16). This is mediated by activation of complex transcriptional machinery within these cells by inflammatory cytokines including GM-CSF, IL-6, G-CSF, IL-1ß, PGE2, TNFα, and VEGF (10–16). Currently known transcriptional regulators of MDSC biology include STAT3, CEBP/β, STAT5, IRF8, S100A8/9, RB, TIPE2 and GCN2 (**Table 1**).

STAT3 is a key repressor of antitumor immunity (39, 40). It impairs antigen presentation and inhibits the production of immunostimulatory cytokines while promoting the expression of immunosuppressive molecules. It is highly active in most cancers where it promotes the production of inflammatory cytokines and growth factors like IL-6, IL-10, IL-23, LIF, VEGF, and HGF (39, 41). These molecules induce STAT3 activation in myeloid precursors which drives cell survival, transcription of immunosuppressive enzymes (ARG1 and iNOS), and aberrant differentiation into MDSCs. It also interacts with C/EBPß at promoter sites to regulate transcription (33, 34). Intriguingly, a decrease in MDSC STAT3 activity in the tumor environment is associated with differentiation into TAMs (42). Within myeloid precursors, STAT3 and STAT5 also inhibit IRF8, a crucial transcription factor for normal myeloid differentiation into monocytes and dendritic cells (23). IRF8 functions as a negative regulator of MDSCs and its downregulation is necessary for pathologic MDSC expansion (23).

S100A8/9 produced by tumors binds to RAGE receptors on myeloid precursors inducing activation of an NF-κB-C/EBPß-STAT3 axis (29). This promotes production of S100A8/9 in MDSCs and drives both expansion and chemotactic migration to tumor sites for immunosuppression. The MDSC-secreted S100A8/9 creates an autocrine feedback loop that exacerbates MDSC accumulation.

High reactive oxygen species (ROS) associated within tumor microenvironments and IL-6 induce TIPE2 in myeloid precursors (30, 31). Active TIPE2 promotes the expression of C/EBPß and STAT3 *via* the PI3K/AKT and MAPK/ERK pathways. This leads to MDSC accumulation and polarization into an immunosuppressive phenotype. In the absence of TIPE2 MDSCs became anti-tumoral indicating TIPE2 functions as a molecular polarity switch in MDSCs (30). GCN2 similarly functions as a polarity switch in MDSCs. It alters myeloid function by inducing C/EBPß and CREB-2/ATF4 which promote MDSC expansion and immunosuppressive activity (32). Epigenetic silencing of Rb by HDAC-2 in myeloid precursors also promotes accumulation of PMN-MDSCs (38).

C/EBPß appears to be an essential player among these effectors in MDSCs.

## C/EBP Protein Family

C/EBPß is the second member of the CCAAT/Enhancer Binding Protein (C/EBP) family of transcription factors (28). C/EBP proteins are basic-region-leucine zipper transcription factors which regulate both emergency and steady state myelopoiesis (35, 43–45). C/EBPα, the first member of the family, regulates steady state myelopoiesis. C/EBPα is highly expressed early identified n the myeloid differentiation process and is an essential molecular switch for the transition from common myeloid precursors to granulocyte macrophage progenitors (46). The role of other C/EBP family proteins, including C/EBPδ and CHOP, are less clear but they are all thought to similarly regulate myelopoiesis as well as modulate the activity of other C/EBP proteins (28). C/EBPδ regulates the expression of inflammatory cytokines including COX-2, iNOS, G-CSF, IL-1β, IL-6, and TNF-α, and has been implicated in MDSC expansion (47, 48). CHOP on the other hand, lacks DNA-binding activity but can form heterodimers with C/EBPß isoforms and other family members, regulating their activity (49). It has similarly been implicated in MDSC expansion *via* these regulatory events (50).

**Figure 1:**
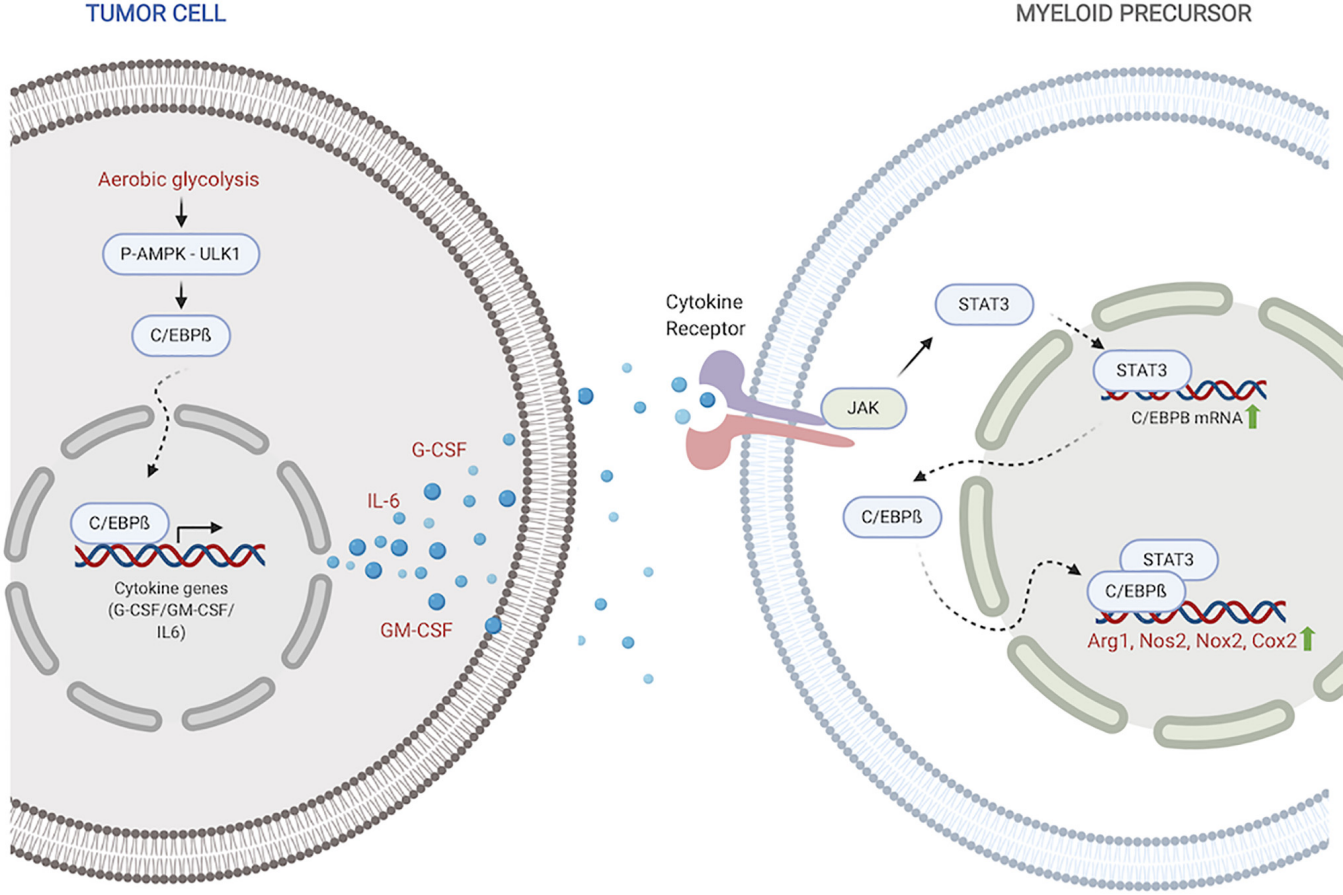
C/EBPß regulates MDSC expansion and function. Within the tumor, C/EBPß promotes transcription of inflammatory cytokines. Inflammatory cytokines then reciprocally induce C/EBPß in myeloid compartment which promotes transcription of immunosuppressive molecules. Created with BioRender.com.

Within the context of MDSC development and function, C/EBPß (also known as IL6-DBP, CRP2, NF-IL6, NF-M or TCF5) is the most important C/EBP (**Figure 1**). It has three isoforms with diverse, context-dependent roles (28, 51). The first two, LAP and LAP*, contain both a DNA-binding domain and an activation domain. The third isoform, LIP, lacks an activation domain and attenuates transcriptional activity *via* heterodimerization with LAP/LAP* (35, 45, 52). C/EBPß controls emergency myelopoiesis, which is a characteristic feature of many solid tumors due to chronic tumor-induced inflammation (53–55). Deregulations of C/EBPß activity are thus a significant contributing factor to aberrant myelopoiesis and MDSC expansion under pathological conditions (27, 28).

Stimulation with inflammatory cytokines like G-CSF, GM-CSF and IL-6 drives an increase in C/EBPß expression and DNA-binding activity (27, 35, 56). Upregulated LAP and LAP* isoforms of C/EBPß function as mediators of cytokine-induced inflammatory response *via* transcriptional activation of inflammatory genes IL-6, TNF and G-CSF, exacerbating the response (45). Under pathological conditions, this sustained inflammatory activation promotes aberrant myeloid development and differentiation into immunosuppressive phenotypes (27, 35, 36). Following IL-6 stimulation, C/EBPß, in concert with STAT3, also promotes miR-21 and miR-181b, which induce NFI-A to promote MDSC accumulation in the bone marrow and spleen (34).

Within tumors, aerobic glycolysis, a hallmark of cancer, leads to an increase in LAP which promotes G-CSF+GM-CSF expression and secretion (37). Li et al. showed that in breast cancer cells, preferential activation of aerobic glycolysis over oxidative phosphorylation, inhibits AMPK-ULK1 and autophagy signaling, allowing stabilization and activity of LAP (37). Cytokines, induced by LAP, travel to the myeloid compartment where they promote expansion of MDSC precursors and direct their differentiation into suppressor cells. Within MDSCs, activated C/EBPß directly binds to and promotes the transcription of immunosuppressive enzymes including Arg1, Nos2, Nox2, and Cox2 (27, 36, 57). These enzymes are crucial members of the MDSC immunosuppressive machinery. Arg1 and Nos2 deplete environmental L-arginine, a crucial amino acid for T-cell survival and anti-tumor activity (58–61). Nox2 increases ROS which block T-cell activation and activity (62, 63). The COX-2-PGE2 cascade suppresses both dendritic and natural killer cell activity, while promoting the expression of immunity repressor PD-L1 (64, 65). It is also plausible that activated C/EBPß in myeloid precursors similarly induces the production of GM-CSF and IL-6 which drive MDSC accumulation and function in autocrine signaling mechanisms.

In macrophages, PI3Kγ activates C/EBPß, which serves as a critical polarization switch from an immunostimulatory to an immunosuppressive phenotype during tumor progression (66). This suggests C/EBPß could also regulate MDSC differentiation into TAMs in the tumor microenvironment.

Seminal work by Marigo et al. showed that C/EBPß deletion in all hematopoietic lineage cells was enough to halt MDSC genesis and completely abrogate their immunosuppressive activity on antigen activated T-cells (27). They also observed significant reduction in both Arg1 and Nos2 expression and activity. C/EBPß deletion potentiated adoptive T-cell therapy resulting in a complete cure for 60% of mice bearing subcutaneous fibrosarcoma. Their work and subsequent studies suggest C/EBPß is an essential mediator of MDSC development and activity (36, 67, 68).

Perhaps unsurprisingly, many studies into molecular effectors of MDSCs have focused on upstream regulators of C/EBPß. Of these recently found effectors, c-Rel, appears to be an essential regulatory partner for C/EBPß in MDSC.

## c-Rel, a New Regulator of MDSC Differentiation and Function

c-Rel, is a member of the NF-κB family of TFs which regulate a variety of molecular processes from embryogenesis to hematopoiesis and inflammation (69, 70). Being a class 2 member of the family, it contains both an N-terminal Rel-homology domain (RHD) and a transactivation domain (TAD) (70, 71). c-Rel’s RHD mediates interactions with other proteins and transcriptional regulators at promoter sites where its TAD recognizes and binds to consensus GGGCTTTCC sequences (69, 72). These interactions, especially with other NF-κB members to form heterodimers, are essential for c-Rel transcriptional activity. c-Rel’s TAD also contains several serine residues which are readily phosphorylated, regulating c-Rel nuclear localization, transactivation and DNA binding activity (73–76).

c-Rel is an important regulator of immune cell function. It is crucial for normal B- and T- cell activation and proliferation (77–81). Upon lymphocyte activation, c-Rel induces IRF-4 in B-cells which promotes cell cycle progression and proliferation. IRF-4 has κB elements in its promoter region to which a c-Rel:p50 heterodimer binds. B-cell proliferation defects have been observed in c-Rel deficient mice (82). Similar defects in T-cell activation and proliferation following stimulation have been observed in c-Rel knockout mice (77).

c-Rel is a key regulator of autoimmunity *via* its role in promoting the generation of Th1, Th17 and Foxp3^+^ regulatory T cells (T_regs_) (83–87). c-Rel is responsible for assembling a transcriptional enhanceosome including RelA, NFAT, SMAD and CREB that binds and transcribes *Foxp3*, a master regulator of T_reg_ immunosuppression (84). c-Rel also directly regulates the expression of many proinflammatory cytokines *via* its context-dependent binding events at promoter sequences (79, 80, 88). Intriguingly, despite its significant roles in both inflammation and autoimmunity, the effects of c-Rel deficiency on immune homeostasis appear to be mostly minor (77).

Although previously thought to primarily function in the lymphoid compartment, mounting evidence suggests a significant role for c-Rel in myeloid cells. We recently showed that c-Rel regulates MDSC expansion and function in cancer (57). Both global and myeloid-specific c-Rel deletion blocked tumor growth and markedly decreased MDSC accumulation in melanoma and lymphoma mice models. The few MDSCs that were generated in the c-Rel knockout mice were defective in suppression when compared to MDSCs from Wild-type mice. c-Rel deletion also altered MDSC metabolism, reducing mitochondrial respiration and glycolysis, inducing a Warburg-like metabolic state. We also observed downregulation of signature MDSC genes in c-Rel knockout mice including Arg1, Nos2, and C/EBPß, key members of the MDSC immunosuppression machinery. There was also heightened inflammatory gene expression in c-Rel deficient MDSCs compared to wild type, a phenotype that was rescued by C/EBPß overexpression. This suggests that c-Rel’s effect in MDSCs is C/EBPß dependent.

Mechanistically, c-Rel directly regulates the transcription of these MDSC signature genes (57). Upon stimulation with GM-CSF and IL-6, c-Rel binds to the promoters of *Arg1* and *Cebpb* where it forms a transcriptional complex with pSTAT3, C/EBPß and p65. ReChIP analyses showed that these factors all bind to the same promoter element, suggesting the formation of a single enhanceosome complex which drives MDSC biology. c-Rel-C/EBPß enhanceosomes have previously been identified as transcriptional regulators in hepatocytes (89, 90).

## Enhanceosomes

Enhanceosomes are high-order protein complexes, usually transcription factors, that bind cooperatively at a gene’s promoter or enhancer regions to activate transcription (91, 92). Many cis-regulatory elements, including promoters and enhancers, contain overlapping DNA binding sites for various transcription factors. This allows the formation of elaborate protein complexes which alter chromatin architecture and recruit the RNA polymerase transcription machinery, regulating gene expression as a functional, nucleoprotein unit (91, 92). These enhanceosome complexes effectively function as “on” and “off” transcriptional switches, specifying key developmental and cell lineage-determining gene regulation events (91, 92). Enhanceosomes could comprise any number of multifunctional transcriptional regulators in an almost limitless number of combinations, specifying the varied cell differentiation programs found in multicellular organisms. An increasing number of enhanceosomes are being described, shifting previously established transcription paradigms.

Fiedler et al. recently described a “Wnt enhanceosome” consisting of ChiLS, Runt/RUNX2, ARID1 and Groucho/TLE which is integrated by Pygo at TCF enhancers to drive Wnt signaling in *Drosophila* (93). Additionally, the Wnt enhanceosome could incorporate a number of factors in a lineage-dependent manner and be switched “off” by Notch. This allows context-dependent regulation of TCF/LEF target genes to simultaneously promote embryogenesis and development while preventing hyperproliferation and cancer. Pawlus et al. similarly described a multifactorial HIF enhanceosome comprising of HIF1, HIF2, RNA poll II and varied transcription factors at enhancer sites for HIF target genes (94). These context-dependent enhanceosomes help explain the dual oncogenic and tumor-suppressive role of HIF-mediate hypoxia. Scotto et al. also showed that multidrug resistance in cancer is governed by an MDR1 enhanceosome at the *MDR1* promoter which can be activated by a variety of stimuli including differentiation agents like retinoic acid, UV radiation and chemotherapy (95). The MDR1 enhanceosome included NF-Y, Sp family transcription factors and histone acetyltransferase PCAF and could be targeted to reverse multidrug resistance.

The assembly and disassembly of enhanceosomes is essential for tight gene regulation in a cell. Because the assembly of a functional enhanceosome complex depends on several factors including local DNA conformation, protein availability and modifications, gene regulation *via* enhanceosomes can be very cell-specific. The absence of any one factor disrupts enhanceosome activity, preventing transactivation. In the case of MDSCs, enhanceosomes at regulatory sites for MDSC signature genes are compelling as key effectors of aberrant MDSC development under pathological conditions.

## The c-Rel-C/EBPß Enhanceosome

**Figure 2:**
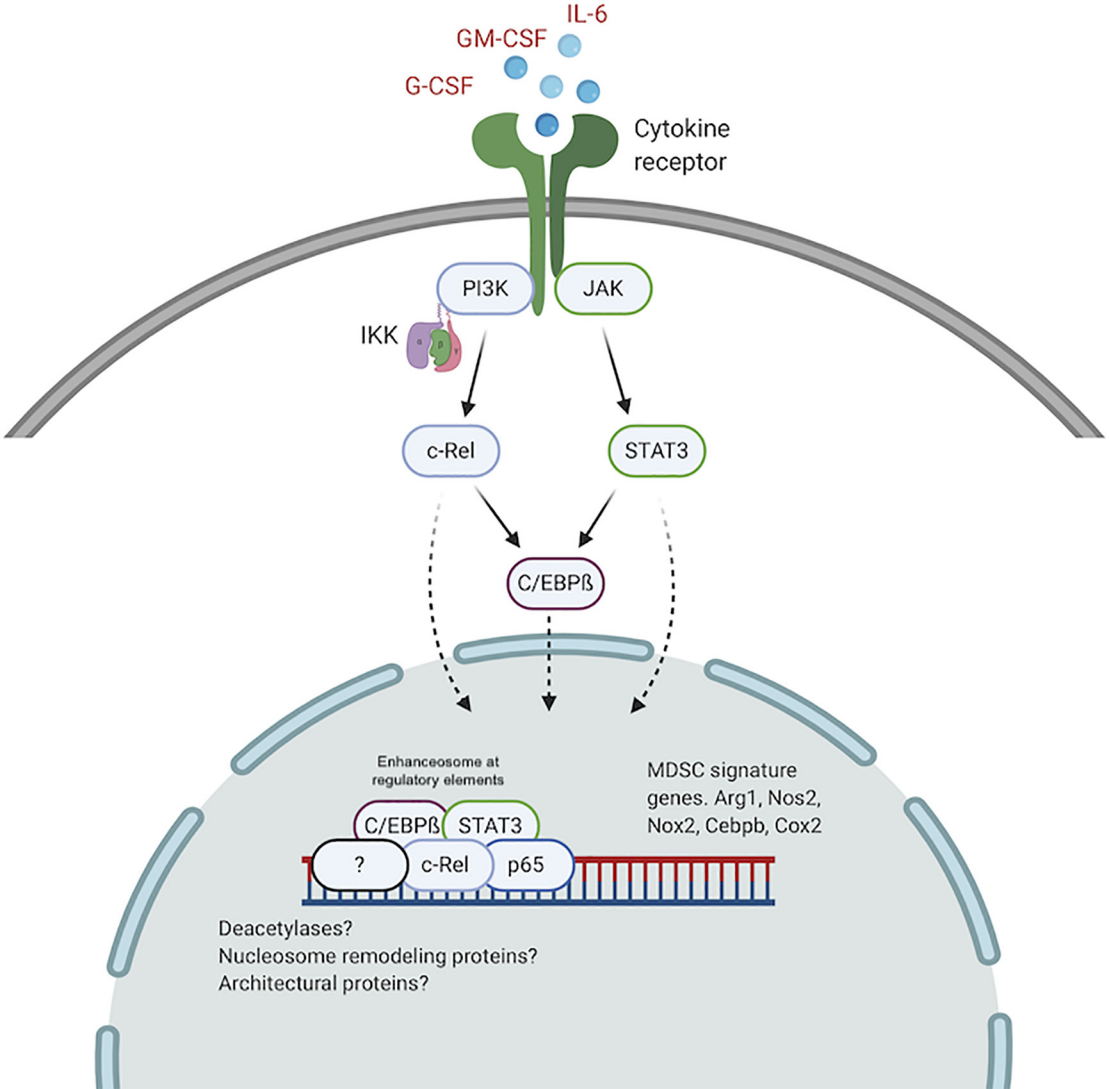
The c-Rel/C/EBPß enhanceosome in MDSCs. c-Rel and C/EBPß induced by tumor secreted cytokines, translocate to the nucleus and assemble an enhanceosome containing STAT3, p65 and other regulators at enhancer sites for immunosuppressive molecules. Created with BioRender.com.

It is plausible that higher levels of active c-Rel and C/EBPß within the nucleus of pathologically activated myeloid cells drive the formation of altered enhanceosomes at regulatory regions for *Arg1, Nos2, Nox2, Cebpb*, and other MDSC genes. Previous work has identified enhanceosomes for several immunosuppressive mediators including Nos2, Arg1, and Nox2 that do not contain either C/EBPß or c-Rel (96–98). We recently showed abundant c-Rel and C/EBPß accumulation at the gene promoters of both *Arg1* and *C/EBPß* following stimulation with GM-CSF and IL-6 (57). In this c-Rel-C/EBPß MDSC enhanceosome model, c-Rel is recruited first to the promoter site and in its absence, the enhanceosome fails to assemble. Following c-Rel binding, pSTAT3, p65 and C/EBPß are recruited to the promoter site to drive transcription and differentiation into immunosuppressive MDSCs (**Figure 2**).

A similar c-Rel-C/EBPß enhanceosome was previously described (89, 90). Cha-Molstad et al. showed that in hepatocytes, cytokine stimulation promotes c-Rel-mediated recruitment of C/EBPß and STAT3 to the *CRP* gene promoter to activate transcription (89). Intriguingly, c-Rel itself was not directly bound to the DNA sequence. c-Rel DNA binding activity is regulated by phosphorylation of the many serine residues within its TAD (73–76). Because we found c-Rel binding to DNA forming the MDSC enhanceosome, it is plausible highly active kinases within pathologically activated myeloid cells contribute to the formation of the MDSC-specific c-Rel enhanceosome. Other post-translational modifications, specific to myeloid cells under pathological activation, that modulate protein-protein interactions and protein-DNA interactions, might drive the formation of MDSC enhanceosomes. Other NF-κB proteins, including p50, have been reported to be involved in MDSC expansion following stimulation by tumor-derived PGE2 (99). We previously showed that c-Rel could bind p50 in MDSCs (57). P50 could similarly be incorporated into the MDSC enhanceosome during tumorigenesis to drive MDSC expansion and activity. The c-Rel-C/EBPß enhanceosome might also contain other nuclear proteins including co-regulators, deacetylases, architectural proteins like HMG I/Y and nucleosome remodeling proteins.

The c-Rel-C/EBPß enhanceosome is also a promising candidate as a biochemical marker for MDSCs. A significant constraint in MDSC research is the lack of reliable markers to characterize this highly heterogeneous cell population (22). Because yields are often low when isolating MDSCs, especially from *in vivo* systems, most studies lack functional validation of immunoregulatory activity. Improved biochemical markers, specific to MDSCs, would provide a simple validatable phenotype for MDSCs. The individual factors within the enhanceosome are not specific to MDSCs: C/EBPß is enriched in monocytes/macrophages (100, 101). c-Rel and p65 are pervasive regulators of B- and T- cell proliferation (77–81). pSTAT3 is a ubiquitous transcription factor within eukaryotic cells (25, 41). However, concurrent activation of all four, as well as other putative members of the enhanceosome, could be indicative of an MDSC phenotype. Monitoring assembly and activation of the c-Rel-C/EBPß enhanceosome could thus be a testable marker for MDSC activation and expansion.

This also provides an exciting therapeutic avenue. We showed that a small molecule inhibitor of c-Rel abrogated MDSC development and immunosuppression *via* disruption of the c-Rel complex (57). Similar approaches targeting individual members, aiming to disrupt their interactions in the MDSC enhanceosome, could have thrilling outcomes. Lee et al. showed that cerulenin, a small molecule inhibitor of the NF-κB enhanceosome in macrophages, might disrupt the assembly of the enhanceosome, suppressing pro-inflammatory activation and sepsis (102). Cerulenin specifically disrupted the p65-TonEBP-p300 complex without affecting their expression or DNA-binding. It had no detectable toxicity and animals could tolerate high doses for several weeks (103). Additionally, our c-Rel inhibitor enhanced the anti-tumor effect of anti-PD-1 antibodies suggesting combinatorial restoration of T cell function (via MDSC inhibition) and activation (via PD-1 inhibition) as a viable clinical strategy (57). The development of a novel class of enhanceosome inhibitors targeting MDSCs could represent an exciting approach to potentiate immunotherapy.

## Conclusion

MDSCs are a product of sustained pathologic inflammation, which develop as a result of aberrant cytokine-mediated activation of complex transcriptional machinery in myeloid precursors (9, 10). They are involved in the pathogenesis of a host of human diseases from cancers to acute infections. In cancer, tumor-produced cytokines mediated by C/EBPß induce c-Rel and C/EBPß in the myeloid compartment, which drives the formation of a c-Rel-C/EBPß-pSTAT3-p65 MDSC enhanceosome. This enhanceosome promotes the transcription of immunosuppressive enzymes and other MDSC signature genes, guiding their differentiation into immunosuppressive cell populations. Because this putative enhanceosome is MDSC-specific, it can be targeted to repress MDSC expansion and immunosuppression. It is thus imperative to further characterize this enhanceosome and develop modalities to inhibit it. Additionally, further studies into other complex transcription programs underlying spatiotemporal gene regulation during aberrant myeloid cell differentiation are warranted. These would identify novel mechanisms and therapeutic targets, which could be blocked clinically to enhance the efficacy of immunotherapies like checkpoint blockade.

## Author Contributions

NF drafted the manuscript and designed the figures. XL and TI reviewed the manuscript structure and science. YC reviewed the manuscript structure, ideas and science. All authors contributed to the article and approved the submitted version.

## Funding

This work was supported in part by grants from the National Institutes of Health (nos. R01-AI152195, R01-AI099216, R01-AI121166, R01-AI143676, and R01-AI136945 to YC); XL was partially supported by grant no. NIH-T32-DK007780.

## Conflict of Interest

The authors declare that the research was conducted in the absence of any commercial or financial relationships that could be construed as a potential conflict of interest.

## Abbreviations

AKT, Protein kinase B; ATF, Activating transcription factor; C/EBPβ, CCAAT-enhancer-binding protein ß; COX-2, Cyclooxygenase 2; CREB, cAMP response element-binding protein; ERK, Extracellular-signal-regulated kinase; G-CSF, Granulocyte colony-stimulating factor; GCN2, General control nonderepressible 2; GM-CSF, Granulocyte-macrophage colony-stimulating factor; HDAC2, Histone Deacetylase 2; HMG I/Y, High mobility group protein; IL-1ß/6/10/23, Interleukin -1ß/6/10/23; IRF8, Interferon Regulatory Factor 8; LAP/LAP*, Liver-enriched activating protein – C/EBPß isoforms; LIF, Leukemia inhibitory factor; LIP, Liver-enriched inhibitory protein; MAPK, Mitogen-activated protein kinase; MDSC, Myeloid-derived suppressor cell; NFAT, Nuclear factor of activated T-cells; NFI-A, Nuclear factor 1 A-type; NOX2, NADPH oxidase 2; PGE2, Prostaglandin E2; PI3K, Phosphoinositide 3-kinase; RAGE, Receptor for advanced glycation endproducts; RB, Retinoblastoma protein; ReCHIP, Re-Chromatin immunoprecipitation; S100A8/9, S100 Calcium Binding Protein A8; STAT3/5, Signal transducer and activator of transcription 3/5; TIPE2, TNF alpha induced protein 8 like 2; TNF, Tumor necrosis factor; VEGF, Vascular endothelial growth factor; c-Rel, Cellular Avian Reticuloendotheliosis Viral Oncogene Homolog; iNOS/NOS2, inducible NO synthase/Nitric Oxide Synthase 2.
